# Interplay between microbial‐derived GABA and host GABA receptor signaling collectively influence the tumorigenic function of GABA in colon cancer

**DOI:** 10.1002/prp2.1226

**Published:** 2024-06-17

**Authors:** Jonathan M. Keane, Philana Fernandes, Florian Kratz, Grace O'Callaghan, Cormac G. M. Gahan, Susan A. Joyce, Catherine Stanton, Niall P. Hyland, Aileen Houston

**Affiliations:** ^1^ APC Microbiome Ireland University College Cork Cork Ireland; ^2^ Department of Medicine University College Cork Cork Ireland; ^3^ School of Microbiology University College Cork Cork Ireland; ^4^ School of Pharmacy University College Cork Cork Ireland; ^5^ School of Biochemistry and Cell Biology University College Cork Cork Ireland; ^6^ Teagasc Food Research Centre Cork Ireland; ^7^ Department of Physiology University College Cork Cork Ireland

**Keywords:** colon tumorigenesis, gamma‐aminobutyric acid, glutamate decarboxylase, inflammation, microbiome, proliferation

## Abstract

Although classically recognized as a neurotransmitter, gamma aminobutyric acid (GABA) has also been identified in colonic tumors. Moreover, the gut microbiome represents another potential source of GABA. Both GABA_A_ and GABA_B_ receptors have been implicated in contributing to the effects of GABA in colorectal cancer, with both pro‐ and anti‐tumorigenic functions identified. However, their subunit composition is often overlooked. Studies to date have not addressed whether the GABA‐producing potential of the microbiome changes over the course of colon tumor development or whether receptor subunit expression patterns are altered in colon cancer. Therefore, we investigated the clusters of orthologous group frequencies of glutamate decarboxylase (GAD) in feces from two murine models of colon cancer and found that the frequency of microbial GAD was significantly decreased early in the tumorigenic process. We also determined that microbial‐derived GABA inhibited proliferation of colon cancer cells in vitro and that this effect of GABA on SW480 cells involved both GABA_A_ and GABA_B_ receptors. GABA also inhibited prostaglandin E_2_ (PGE_2_)‐induced proliferation and interleukin‐6 (IL‐6) expression in these cells. Gene expression correlations were assessed using the “Cancer Exploration” suite of the TIMER2.0 web tool and identified that GABA receptor subunits were differentially expressed in human colon cancer. Moreover, GABA_A_ receptor subunits were predominantly positively associated with PGE_2_ synthase, cyclooxygenase‐2 and IL‐6. Collectively, these data demonstrate decreased potential of the microbiome to produce GABA during tumorigenesis, a novel anti‐tumorigenic pathway for GABA, and that GABA receptor subunit expression adds a further layer of complexity to GABAergic signaling in colon cancer.

## Graphical abstract

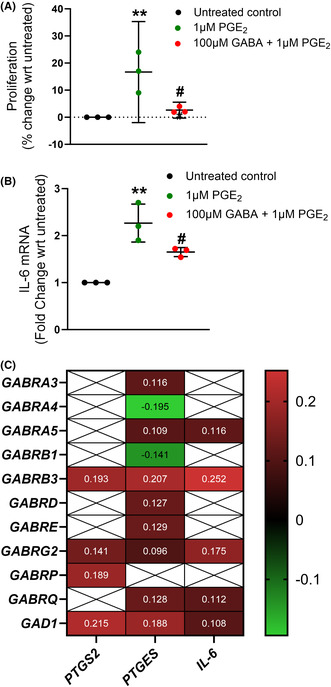

**Abbreviations**
AOM: azoxymethane
COAD: colon adenocarcinoma
COG: clusters of orthologous group
COX‐2: cyclooxygenase‐2
FCS: fetal calf serum
FU: fluorescence units
GAD: glutamate decarboxylase
IL‐6: interleukin‐6
MSG: monosodium glutamate
PGE_2_
: prostaglandin E_2_

## 1 INTRODUCTION

Gamma aminobutyric acid (GABA) is primarily known as an inhibitory neurotransmitter. However, it is also a bacterial‐derived metabolite which has been shown to have potential tumor modifying effects, with both pro‐ and anti‐tumorigenic functions identified.^1^ Although both GABA_A_ and GABA_B_ receptors have been implicated in mediating these effects, their subunit composition is often overlooked. In particular, GABA_A_ receptors consist of an assembly of multiple subunits (α1–6, β1–3, γ1–3, δ, ϵ, θ, π, ρ1–3) that form a pentamer, whereas the GABA_B_ receptor is composed of two main subunits, termed GABABR1 and GABABR2. Moreover, studies to date have not addressed whether the GABA‐producing potential of the gut microbiome changes over the course of colon tumor development or whether the receptor subunit expression patterns are altered in human colon cancer.

Anti‐tumor activity of the gut microbiome has been attributed to several factors such as production of bacterial metabolites and their effects on the immune response.^2^ Harnessing the ability of probiotics and microbial‐targeted interventions represents a potential anti‐tumorigenic strategy in colon cancer.^3^ Moreover, tumorigenic processes, such as proliferation, have also been shown to be regulated by inflammatory pathways, one of which is the cyclooxygenase (COX)‐2 signaling pathway.^4^ COX‐2 is activated in response to inflammatory stimuli, with prostaglandin E_2_ (PGE_2_) shown to mediate its proinflammatory and tumor‐promoting effects in colon cancer.^4^ However, whether crosstalk between GABA and PGE_2_ occurs in colon cancer is unexplored.

## 2 MATERIALS AND METHODS

### 2.1 Animal studies and microbiome sequencing

C57BL/6J female mice (6–8 weeks of age) were obtained from Envigo (Blackthorne, UK) and Apc^Min/+^ female mice on a C57BL/6J background were acquired from Charles River (Kent, UK) at 4 weeks of age. Mice were housed in a specific pathogen‐free facility on a 12‐h light/dark cycle at 22°C with access to water and chow ad libitum. After acclimatization, for the chemically induced azoxymethane (AOM) model of colon cancer, mice were administered an intraperitoneal injection of 10 mg/kg AOM each week for five consecutive weeks and control mice received phosphate buffer saline.^5^ Fecal samples were collected at 8, 12, 24, and 48 weeks. For the hereditary Apc^Min/+^ model of colon cancer, C57BL/6J mice and Apc^Min/+^ mice were age‐matched and fecal samples collected at 4, 7, 11, and 14 weeks of age.

Fecal 16S rRNA sequencing DNA was extracted from feces using the QIAamp Fast DNA Stool Kit as per the manufacturer's instructions with the addition of a bead‐beating step. The V3‐V4 variable region of the 16S rRNA gene was amplified from each extracted DNA sample according to the 16S metagenomic sequencing library protocol (Illumina, San Diego, CA, USA) and sequenced on an Illumina MiSeq.^5^

### 2.2 Maintenance and culture of *Levilactobacillus brevis* (previously known as *Lactobacillus brevis*) DPC6108

*Levilactobacillus brevis* DPC6108 used in this study was maintained in the Teagasc Moorepark Food Research Centre culture collection and cultured in MRS broth (Difco, MI, USA) supplemented with 0.05% (w/v) l‐cysteine‐hydrochloride (mMRS) (98% pure; Sigma Chemical Co., MO, USA) under anaerobic conditions at 37°C. To determine the ability of *L. brevis* DPC6108 to convert monosodium glutamate (MSG) to GABA, *L. brevis* DPC6108 was cultured in mMRS supplemented with MSG (30 mg/mL). Conversion of MSG to GABA was determined by analyzing the free amino acid content.^6^ In the presence of MSG, the GABA concentration produced by this strain was 0.858 mg/mg which represents a working concentration of 1 μM GABA in this study. In the absence of MSG, the GABA concentration was 0.015 mg/mg.

### 2.3 Cell lines and culture conditions

SW480 and CT26 colonic tumor cells were obtained from the American Type Culture Collection (Rockville, MD, USA). Cells were maintained in DMEM containing 10% fetal calf serum (FCS) and penicillin–streptomycin at 37°C with 5% CO_2_ in a fully humidified atmosphere. Cells were seeded at 1 × 10^5^ cells/mL unless otherwise stated and cultured overnight.

### 2.4 Proliferation assay

Cells were seeded in six‐well plates, then treated with either bacterial‐derived GABA (1 μM; 16 h) or exogenous GABA (1–100 μM; 24 h). For the GABA receptor antagonist studies, cells were pretreated with vehicle, phaclofen (100 μM; 1 h) or bicuculline (100 μM; 1 h) prior to the addition of GABA. To examine the effects of exogenous GABA on PGE_2_‐induced proliferation, cells were stimulated for 24 h with GABA (100 μM) and PGE_2_ (1 μM). Cells were then washed, media supplemented with 44 μM resazurin was added, and resazurin reduction to resorufin measured fluorometrically using a GENios plate reader (TECAN, Grodig, Austria) and Xfluor spreadsheet software. Results obtained were expressed in fluorescence units (FU) and the percentage viability calculated as follows: (FU‐treated/FU untreated control) × 100. Values were normalized relative to the control cells.

### 2.5 Migration assay

SW480 cells were seeded in serum‐free media in Transwell inserts (8 μM pore size). Inserts were placed into 24‐well plates containing cell culture media (10% FCS). Cells were incubated in the presence of GABA (1 μM and 100 μM; 16 h) and migration assessed using a Millipore Colorimetric Migration assay. The migration of cells from serum‐free media to 10% serum served as the positive control.

### 2.6 Real‐time RT‐PCR

RNA was isolated from cells and colonic tissues and reverse transcribed to cDNA. Real‐time RT‐PCR was performed using an ABI PRISM 7500 Sequence Detection System. All samples were run in triplicate, and relative quantitation was calculated using the 2^−ΔΔCt^ method. Transcript levels were normalized to the amount of β‐actin mRNA, and expression levels are shown as fold induction relative to control.

### 2.7 Colorectal adenocarcinoma and normal tissue gene expression

Gene expression comparison of GABA_A_ and GABA_B_ receptor subunits, GAD1, PTGS2, PTGES, and interleukin (IL)‐6 expression was assessed using the “Cancer Exploration” suite of the TIMER2.0 web tool.

### 2.8 Statistical analysis

Clusters of orthologous group (COG) frequency, proliferation, migration, and gene expression data were analyzed by one‐way ANOVA with a Šídák's multiple comparisons test. In Table 1, statistical significance was computed by the Wilcoxon test. The Cox proportional hazard model was used to evaluate the outcome significance of gene expression. The numbers in the heatmap in Figure 2C represent the spearman's rho correlation. Data are presented as mean ± SD or with 95% confidence intervals where data are presented as percentage change. *p* < .05 was considered significant.

**TABLE 1:** Expression of GABA receptor subunit genes and clinical relevance of gene expression in colon adenocarcinoma (COAD).

GABA receptor gene	Expression in COAD	Clinical Outcome (Z‐score)
*GABRA1*	*p* < .001, nd	4.284, *p* < .001
*GABRA2*	*p* < .001, ↓	0.305, *p* = ns
*GABRA3*	*p* < .001, nd	1.474, *p* = ns
*GABRA4*	*p* < .05, nd	−1.515, *p* = ns
*GABRA5*	*p* < .05, nd	4.027, *p* < .001
*GABRA6*	ns, ↔	0.083, *p* = ns
*GABRB1*	*p* < .001, ↑	−1.055, *p* = ns
*GABRB2*	*p* < .001, ↓	0.313, *p* = ns
*GABRB3*	*p* < .001, ↓	−0.508, *p* = ns
*GABRD*	*p* < .001, ↑	4.636, *p* < .001
*GABRE*	*p* < .001, ↑	2.127, *p* < .05
*GABRG1*	*p* < .001, nd	2.777, *p* < .01
*GABRG2*	*p* < .001, ↓	−0.963, *p* = ns
*GABRG3*	ns, ↔	3.852, *p* < .001
*GABRP*	*p* < .001, ↑	1.602, *p* = ns
*GABRQ*	ns, ↔	1.815, *p* = ns
*GABBR1*	ns, ↔	4.687, *p* < .001
*GABBR2*	ns, ↔	2.688, *p* < .01

*Note*: COAD tumors were analyzed by TIMER 2.0 (http://timer.cistrome.org/database) for differential expression of GABA_A_ and GABA_B_ receptor subunits between colorectal tumors (*n* = 457) and adjacent normal tissues (*n* = 41). Statistical significance was computed by the Wilcoxon test. The Cox proportional hazard model was used to evaluate the outcome significance of gene expression. nd, could not be determined from the expression data analysis in TIMER 2.0.

Abbreviations: GABA, gamma aminobutyric acid; COAD, colorectal adenocarcinoma; ns, non‐significant.

### 2.9 Nomenclature of targets and ligands

Key protein targets and ligands in this article are hyperlinked to corresponding entries in http://www.guidetopharmacology.org, the common portal for data from the IUPHAR/BPS Guide to PHARMACOLOGY,^7^ and are permanently archived in the Concise Guide to PHARMACOLOGY 2019/20.^8^, ^9^

## 3 RESULTS

**FIGURE 1:**
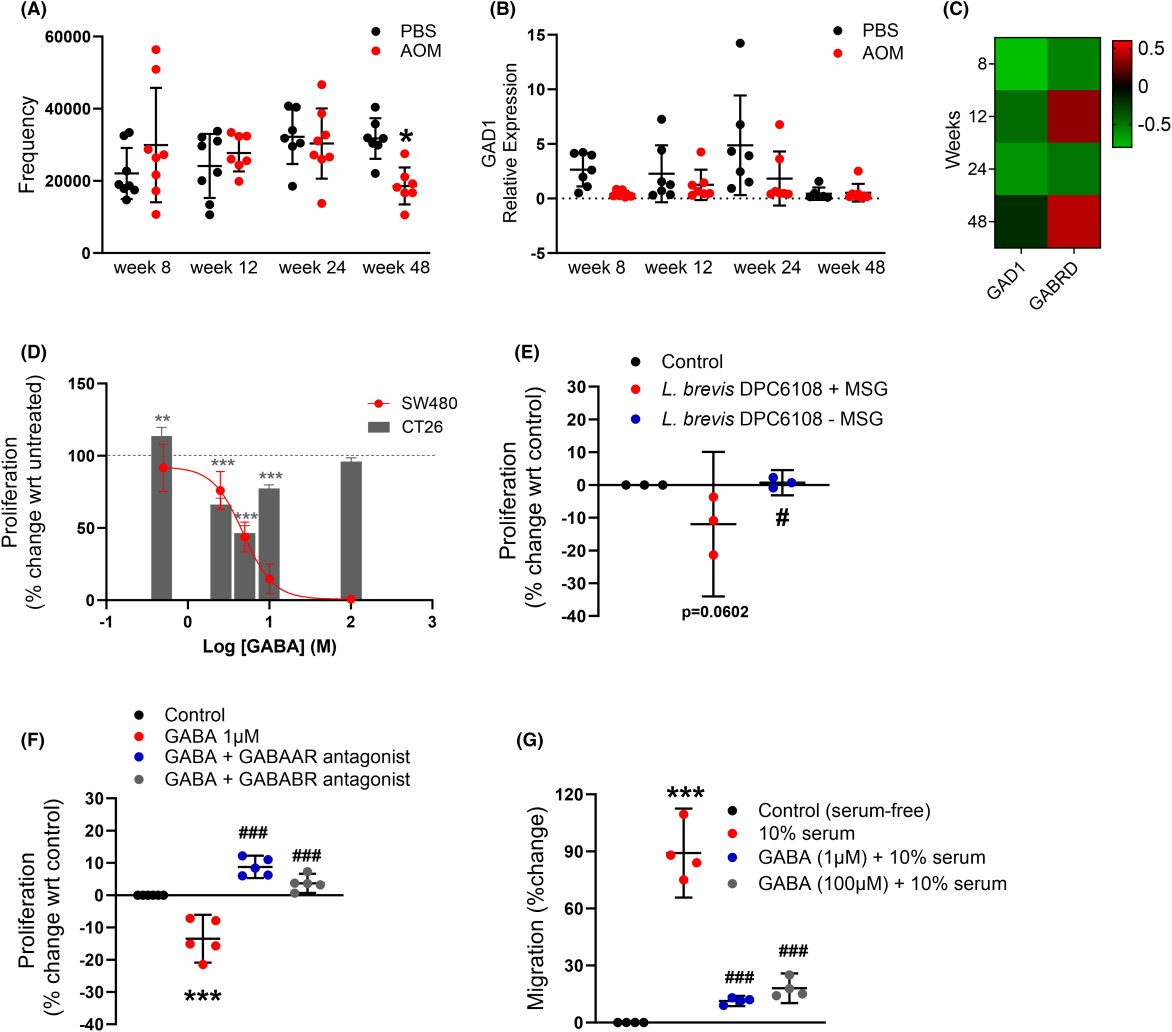
Microbial GAD expression is altered early in the AOM model of colon cancer and GABA has inhibitory effects on proliferation and migration of colon cancer cells in vitro. The frequency of the GAD gene that converts glutamate to GABA is significantly decreased early in the tumorigenic process at the time at which adenomas are developing in the AOM model of colon cancer (A; *n* = 7–8 mice; *F*(7, 52) = 2.261, *p = .0436). However, expression of host GAD1 did not significantly change over the course of tumorigenesis (B; *n* = 6–8 mice). In terms of fold‐change there were no differences in the expression of either GAD1 or GABRD relative to the control group over time. (C; *n* = 6–8 mice). Exogenous GABA significantly altered the proliferation of SW480 and CT26 cells in a concentration‐dependent manner (D; *n* = 3; for CT26, **p = .0028, ***p < .0001). The anti‐proliferative effect of *Levilactobacillus brevis* DPC6108 was significantly inhibited when grown in the absence of MSG compared to when grown in the presence of MSG (E; *n* = 3 biological replicates; ^#^ *p* = .0488). Moreover, the anti‐proliferative effect of GABA (***p < .0001) was significantly inhibited by both GABA_A_ and GABA_B_ receptor antagonists (F; *n* = 5–6; 3 biological replicates; ^###^ *p* < .0001). Cells significantly migrated in the presence of 10% serum (G; *n* = 4 technical replicates; ***p < .001) and both 1 μM and 100 μM GABA significantly suppressed the ability of SW480 cells to migrate toward 10% serum (G; *n* = 4 technical replicates; ^###^ *p* < .001). All data were analyzed by one‐way ANOVA followed by Šídák's multiple comparisons test. GABA, gamma aminobutyric acid; GAD, glutamate decarboxylase; MSG, monosodium glutamate.

COG frequencies of GAD, a key enzyme involved in the synthesis of microbial GABA, at different timepoints were determined in two different models of colon cancer. In a model of chemically‐induced sporadic colon cancer, the AOM model, frequencies of this COG were significantly decreased in AOM‐treated mice at Week 48 relative to PBS‐treated controls (Figure 1A), which was the timepoint at which adenomas were first detectable.^5^ In contrast the expression of host GAD1 was unchanged at week 48 (Figure 1B,C). In a genetic model of colon cancer, the APC^Min/+^ mouse model, frequencies of this COG were also reduced, but not significantly, at Week 7 relative to the parental strain (Figure S1).

In support of a potential inhibitory role for GABA in tumorigenesis,^10^ exogenous GABA significantly suppressed cellular proliferation in SW480 cells in a concentration‐dependent manner with an IC_80_ value of 8.6 μM (5.7–15.6 μM; Figure 1D). While GABA also had an inhibitory effect on murine CT26 colon tumor cells, this inhibitory effect was only observed at concentrations of GABA between 2.5 and 10 μM, with the lowest concentration (500 nM) examined inducing proliferation.

We next examined the effects of GABA‐rich supernatants derived from a GABA‐producing strain, *L. brevis* DPC6108, on colon cancer cell proliferation. *L. brevis* DPC6108 was grown in the presence of MSG to stimulate the production of GABA. This supernatant significantly suppressed proliferation in SW480 human cancer cells relative to *L. brevis* DPC6108 grown in the absence of MSG (control for GABA production; Figure 1E). Given that proliferation was unaffected in the absence of MSG, this would suggest that the inhibitory effect of the supernatant was due to GABA, and not other factors present in the supernatant.

To determine which receptor was responsible for the anti‐proliferative effect of GABA, we applied a pharmacological approach using GABA_A_ or GABA_B_ receptor antagonists. The anti‐proliferative effect of exogenous GABA was significantly inhibited by antagonism of either the GABA_A_ or the GABA_B_ receptor (Figure 1F). As well as activating different signaling pathways, GABA_A_ and GABA_B_ receptors are comprised of different receptor subunits. Using the web tool TIMER2.0 to assess RNAseq gene expression data from human colon adenocarcinomas, expression of the GABA_A_ receptor subunits, but not the GABA_B_ receptor subunits, was found to be differentially expressed in normal versus colon tumor tissue (Table 1). However, irrespective of whether expression of GABA_A_ or GABA_B_ receptor subunits were altered in colon cancer, expression of particular receptor subtypes (GABRA1, GABRA5, GABRD, GABRE, GABRG1, GABRG3, GABBR1, and GABBR2) was significantly associated with poor clinical outcome (Table 1). Given that GABRD is associated with poor prognosis in colon cancer,^11^ we examined the expression of this particular GABA_A_ receptor subunit over time in the AOM mouse model (Figure 1C). Although expression changed during colon tumor development, this did not reach statistical significance. One factor contributing to poor clinical outcome is the presence of metastases, with migration representing an important step in the metastatic process. In this regard, we also observed an inhibitory effect of GABA on migration of SW480 cells (Figure 1G). This anti‐migratory effect appears to be mediated through GABA_B_ and not GABA_A_ receptor activation.^12^, ^13^

**FIGURE 2:**
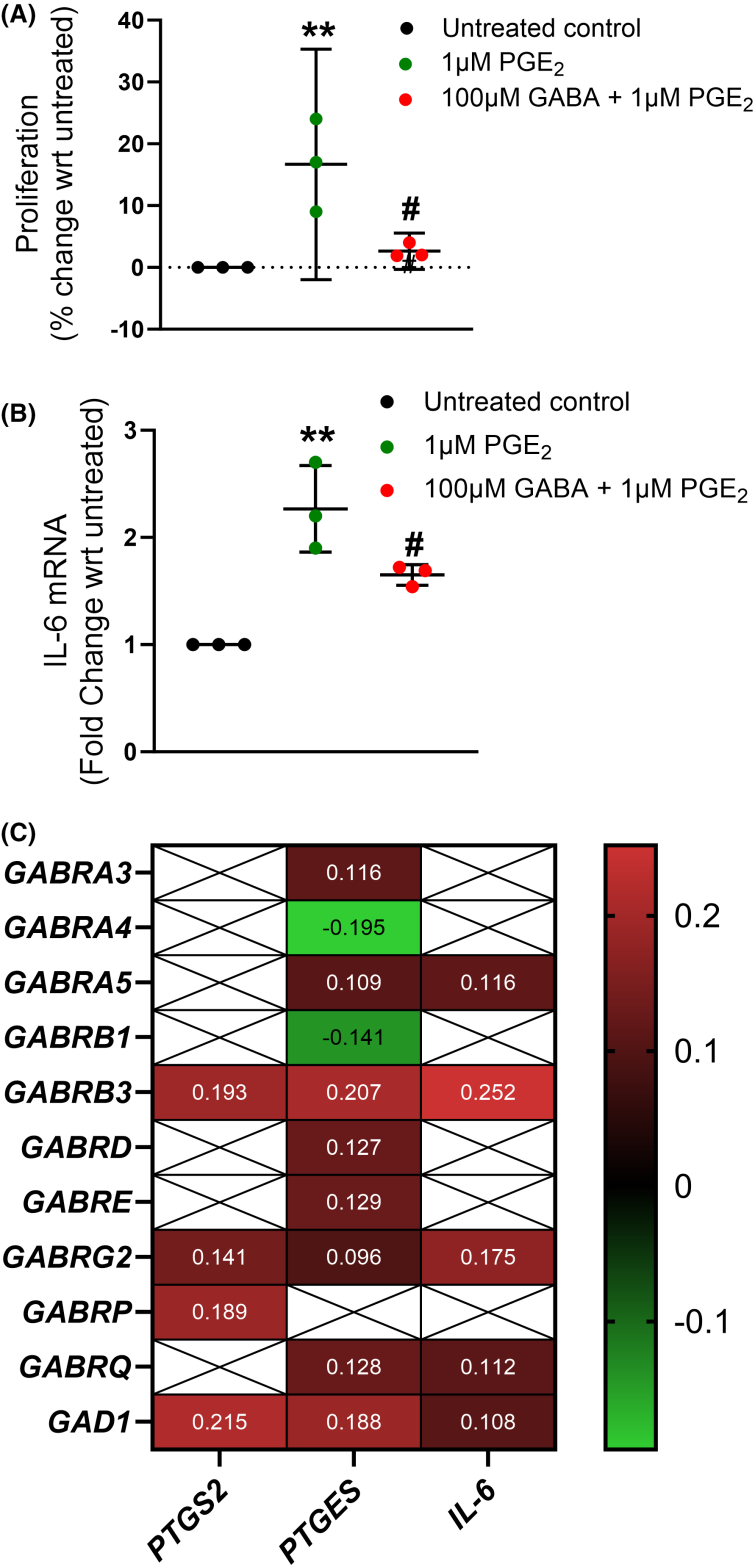
Evidence of crosstalk between GABA and PGE_2_ in colon cancer. PGE_2_ significantly increased proliferation of colon cancer cells (A; *n* = 3 technical replicates; **p = .0070; Šídák's multiple comparisons test), with GABA significantly suppressing this induction (^#^ *p* = .0156). Expression of IL‐6 was increased by PGE_2_ (B; *n* = 3 technical replicates; **p = .0013; Šídák's multiple comparisons test) and this was inhibited by GABA (^#^ *p* = .0393). TIMER2.0 was used to explore the relationship between GABA_A_ receptor subunit expression and GAD1 with genes associated with PGE_2_ production (PTGS2 and PTGES) and IL‐6 (C; Spearman's rho correlation). Filled boxes represent significant associations and white boxes indicate the absence of an association. Red indicates positive associations and green indicates negative associations. GABA, gamma aminobutyric acid; GAD, glutamate decarboxylase; IL‐6, interleukin‐6; PGE_2_, prostaglandin E_2_.

Finally, to investigate the crosstalk between GABA and inflammatory pathways implicated in colon carcinogenesis, we investigated the effect of GABA on PGE_2_ signaling in colon cancer cells. PGE_2_ significantly increased the proliferation of SW480 cells, and this was significantly inhibited by GABA (Figure 2A). GABA also significantly suppressed PGE_2_‐induced expression of the cytokine IL‐6 (Figure 2B). Gene expression of GABRB3, GABRG2, and GAD1 positively correlated with genes involved in the synthesis of PGE_2_ (PTGS2 and PTGES) as well as IL‐6 (Figure 2C) in human colon cancer.

## 4 DISCUSSION

Although several studies report an inhibitory effect of GABA on proliferation of colon cancer cells, the data are not consistent in this regard. While we demonstrated that GABA inhibited proliferation with an IC_80_ of approximately 8 μM, a recent study showed that the inhibition of GABA at concentrations 10‐fold less than this had a pro‐proliferative effect.^14^ Moreover, our data would also suggest that different colon cancer cell lines can respond differently to the same concentrations of GABA in vitro which may account for some of the discrepancies reported to date. Here, we also show that the gut microbiome not only represents another potential source of GABA but also that the GABA‐producing potential of the microbiome changes over the course of colon carcinogenesis. Of note, this GABA‐producing potential is significantly decreased at a time when adenomas first develop,^5^ suggesting that GABA has an inhibitory effect early in the tumorigenic process. We and others^10^ have demonstrated that exogenous GABA can inhibit proliferation of colon cancer cells at concentrations 10‐fold or higher than that secreted by tumor cells.^14^ This anti‐proliferative effect may be dependent on the presence of functional GAD, as in the absence of GAD1, higher concentrations of GABA promoted cancer cell growth.^14^ Although not significant, over the course of tumorigenesis in the AOM model we observed a decrease in the expression of host GAD1 which appeared to normalize at the time at which microbial GAD was decreased. These data suggest that both host and microbial‐derived GABA may play different roles in cancer and point toward the importance of the source and concentration of GABA in determining the tumorigenic effects of GABA, at least in the context of colon cancer.

The anti‐proliferative effects we observed were sensitive to inhibition of either GABA_A_ or GABA_B_ receptors. While we also observed an inhibitory effect on migration, we did not investigate which GABA receptor was involved, given that GABA_B_ receptors have been shown to mediate this process.^12^, ^13^ As activation of GABA receptors impacts multiple signaling pathways,^1^ the inhibitory effects of the antagonists on proliferation could be facilitated by different cellular mechanisms. For example, GABA has been shown to both activate and inhibit the AKT signaling pathway, which in turn can differentially influence the response of tumor cells to GABA.^1^ As well as activating different signaling pathways, GABA_A_ and GABA_B_ receptors are also comprised of different receptor subunits giving rise to further complexity. Recent studies in different cancers have shown that the GABA_A_ receptor subunit composition can impact the effect of GABA. For instance, in hepatic carcinoma, suppressing the GABA_A_R_Q_ and GABA_A_R_A3_ subunits resulted in reduced tumor growth in vitro and in vivo.^15^ In contrast, suppressing the GABA_A_R_B3_ subunit had the opposite effect.^15^ Given that GABA_A_ receptor subunit expression was either increased or decreased in colon cancer (Table 1), the subunit composition of the receptor likely plays an important role in determining the function of GABA in colon tumorigenesis. For example, GABRD, a subunit of the GABA_A_ receptor, promotes proliferation and predicts poor prognosis in colon cancer.^11^ This concurs with our observation that GABRD expression is increased in colon cancer and correlates with poor clinical outcome. While we did not see any significant change in the expression of GABRD in our mouse model of colon cancer, our study concluded at the point of adenoma formation,^5^ with changes in expression potentially occurring later in the tumorigenic process. Our data further identify that other GABA_A_ receptor subunits in particular are differentially expressed in colon cancer, but that both GABA_A_ and GABA_B_ receptor subunits are associated with clinical outcome.

Our findings also provide mechanistic insight into the crosstalk between GABA and inflammatory signaling pathways implicated in colon cancer, in particular the COX‐2 and PGE_2_ pathway. Consistent with these findings, GABA has been shown to augment the anti‐tumorigenic effect of the COX‐2 inhibitor, celecoxib, in a murine model of pancreatic cancer.^16^ In further support of this crosstalk, we identified a significant correlation between expression of the GABA_A_ receptor subunits GABRB3 and GABRG2 and genes encoding COX‐2, PGE synthase and IL‐6. These two GABA_A_ receptor subunits have also been shown to correlate with the occurrence of colon cancer.^17^ Finally, IL‐6 has been reported to be a crucial tumor‐promoting cytokine with expression significantly increased in colorectal carcinoma.^18^

## 5 CONCLUSION

This is the first study to demonstrate that microbial GABA production could change during the course of tumorigenesis in a mouse model of colon cancer. However, a limitation of the COG analysis is that it only infers gene function and may not reflect fecal GABA concentrations in vivo. While the anti‐proliferative effect of *L. brevis* DPC6108 on colon tumor cells in vitro supports a role for GABA‐producing microbes as potential therapeutic interventions, future in vivo studies are now warranted to validate this approach, potentially at times when microbial production is compromised. Moreover, GABA receptor subunit expression in human colon cancer reveals a further layer of complexity in GABAergic signaling which needs to be considered in future studies deciphering the role of GABA not only in colon cancer but also in different types of cancer.

## AUTHOR CONTRIBUTIONS

Conception and design: Cormac G. M. Gahan, Susan A. Joyce, Catherine Stanton, Niall P. Hyland and Aileen Houston. Acquisition of data: Jonathan M. Keane, Philana Fernandes, Florian Kratz, Grace O'Callaghan. Analysis and interpretation of data: Jonathan M. Keane, Philana Fernandes, Florian Kratz, Grace O'Callaghan, Niall P. Hyland and Aileen Houston. Drafting the manuscript: Niall P. Hyland and Aileen Houston. Final approval of the version to be published: Jonathan M. Keane, Philana Fernandes, Florian Kratz, Grace O'Callaghan, Cormac G. M. Gahan, Susan A. Joyce, Catherine Stanton, Niall P. Hyland and Aileen Houston.

## FUNDING INFORMATION

This study was supported by Science Foundation Ireland through a Centre Award to APC Microbiome Ireland (12/RC/2273_P2).

## CONFLICT OF INTEREST STATEMENT

The authors are not aware of any conflicts of interest that might be perceived as affecting the findings of this study.

## ETHICS STATEMENT

Animal experiments were conducted in accordance with the regulations and guidelines of the Irish Department of Health following approval by the University College Cork Animal Experimentation Ethics Committee (2011/023).

## Supporting information


Figure S1.

## Data Availability

The data that support the findings of this study are available from the corresponding author upon reasonable request.
