# Supplementary figures and images for: Interplay between microbial‐derived GABA and host GABA receptor signaling collectively influence the tumorigenic function of GABA in colon cancer
Source: Pharmacol Res Perspect. 2024 Jun 17;12(4):e1226. doi: 10.1002/prp2.1226 (PMC11182776; doi:10.1002/prp2.1226)

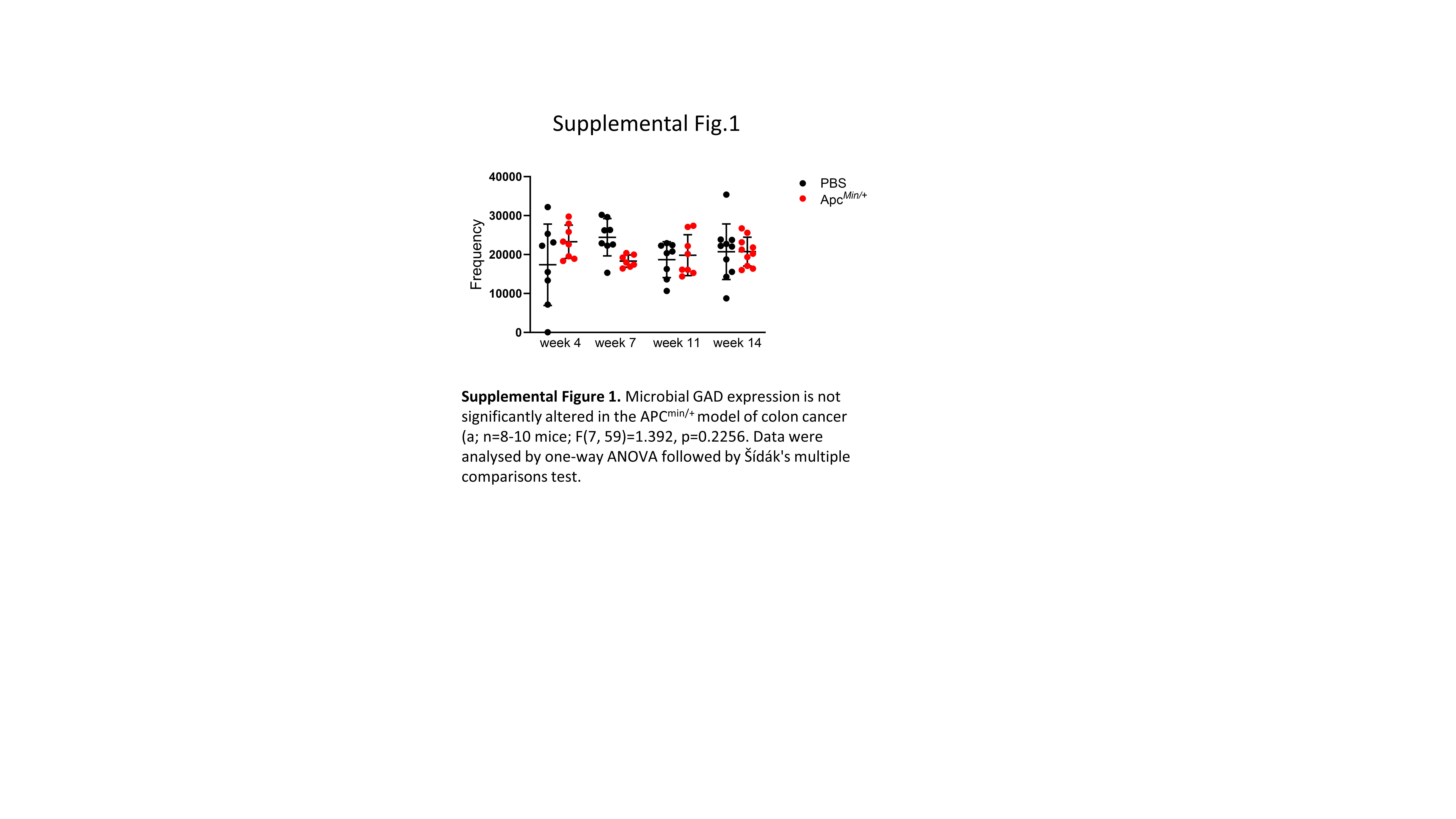

Supplement: Supplementary file 1 — Figure S1. [file PRP2-12-e1226-s001.tif]
